# Large concealed paravaginal haematoma: A case report of an occult postpartum haemorrhage

**DOI:** 10.1016/j.crwh.2021.e00311

**Published:** 2021-03-26

**Authors:** William Stobie, Danendran Krishnan

**Affiliations:** Department of Obstetrics & Gynaecology, Sutherland Hospital, Caringbah, NSW 2232, Australia

**Keywords:** Paravaginal hematoma, Postpartum haemorrhage, Conservative management

## Abstract

Paravaginal haematomas are common but rarely do they cause severe postpartum haemorrhage. While surgical evacuation is classically recommended for large haematomas, conservative management may be an appropriate strategy.

We present the case of a 30-year-old primiparous woman with a large paravaginal haematoma causing a postpartum haemorrhage managed conservatively.

The patient became hypotensive to 80/40 mmHg three and a half hours after forceps delivery, despite minimal vaginal bleeding. On examination she had a palpable mass at the right vaginal wall and uterine fundus deviated to the right, above the umbilicus. A computerised tomography examination of the pelvis demonstrated two paravaginal haematomas, the largest measuring 7 cm × 8 cm × 12 cm, extending superiorly into the supralevator and extraperitoneal space. The patient's haemoglobin fell from 13.2 g/dL to 7.1 g/dL. She was managed conservatively with analgesia, intravenous fluid resuscitation and one unit of packed red blood cells, and was discharged home three days postpartum.

This case supports the conservative management of even large paravaginal haematomas, which may extend into the supralevator space, making surgical evacuation technically difficult.

## Highlights

• Paravaginal haematomas occur from venous bleeding deep to the vaginal mucosa.
• They may extend superiorly into the supralevator and extraperitoneal space.
• Surgical evacuation may be complicated by the difficulty in accessing this space.
• Computerised tomography imaging of suspected large paravaginal haematomas may guide management.
• Conservative management, whereby the haematoma self-tamponades, is a viable option.

## 1 Introduction

Paravaginal haematomas are common but are rarely large enough to cause severe postpartum haemorrhage. Typically, they arise following trauma from the fetal head, shoulders, internal manoeuvres or instrumental delivery and are recognised through symptoms of pain, swelling or pressure and signs of a unilateral palpable mass or fluctuant swelling. Their incidence varies with the methods used to diagnose them. Denson et al. [1] demonstrated small pelvic floor haematomas of 1 mL or more in 26% of women following vaginal delivery with routine endovaginal ultrasound in all women. Conversely, a retrospective Swedish population-based study identified a vaginal haematoma rate of 0.8 per 1000 births [2].

A large potential space exists adjacent to the mucosa of the vaginal wall, extending from the vulva inferiorly to the extraperitoneal space superiorly, bounded by the broad ligament and other peritoneal reflections onto the bladder, uterus and rectum.

As demonstrated in this case, the capacity of this space can conceal a large haematoma, which may be the main source of a severe postpartum haemorrhage, even in the absence of significant vaginal bleeding. Of concern is that this may occur insidiously and in the absence of any symptoms preceding hypovolaemia, especially in the setting of neuraxial anaesthesia.

## 2 Case presentation

We present the case of a large concealed paravaginal haematoma as a cause of severe postpartum haemorrhage.

A 30-year-old primiparous woman presented at 40 weeks and 1 day of gestation in early labour. Her pregnancy was complicated by the diagnosis of a small for gestational age baby (abdominal circumference 5th percentile, estimated fetal weight 3260 g [26th centile]) on ultrasound at 39 weeks and 5 days. Otherwise she had an uncomplicated antenatal course.

The patient received an epidural block and augmentation of labour with oxytocin and progressed well in labour with a 1st stage of 5 h and 45 min and 2nd stage of 35 min.

A fetal bradycardia was noted on cardiotocography at full cervical dilatation and a live male infant weighing 2740 g was delivered by Neville Barnes forceps in one pull, positioned directly occipito-anterior from the level of the ischial spines. The baby had Apgar scores of 8 at one minute and 9 at five minutes. A right mediolateral episiotomy was made and repaired without complication. Estimated blood loss was 400mLs immediately postpartum and mother and baby were well.

3.5 h postpartum the patient was hypotensive, with a BP of 80/40 mmHg, and heart rate of 94 bpm despite 1 L of intravenous fluid resuscitation and minimal vaginal bleeding postpartum. She complained of worsening rectal pain despite being given oxycodone 5 mg and morphine 10 mg subcutaneously. On examination the uterine fundus was deviated to the right and palpable well above the umbilicus. On vaginal examination there was a large palpable mass extending up the right vaginal wall. Repeat haemoglobin was 7.1 g/dL, down from 13.2 g/dL intrapartum, with normal platelets and coagulation studies.

**Fig. 1:**
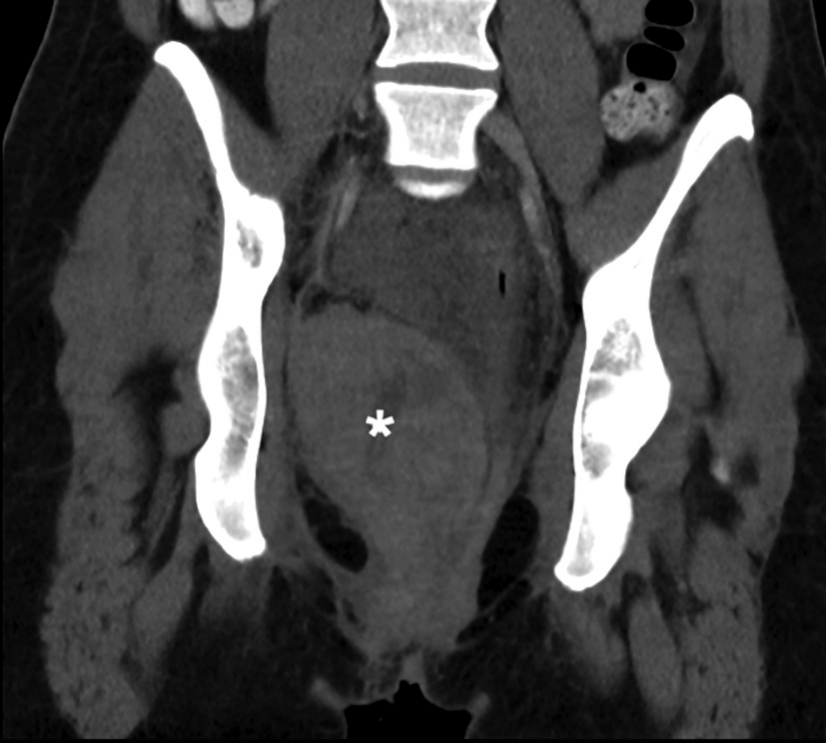
CT scan showing a large paravaginal haematoma extending to the supralevator space*.

**Fig. 2:**
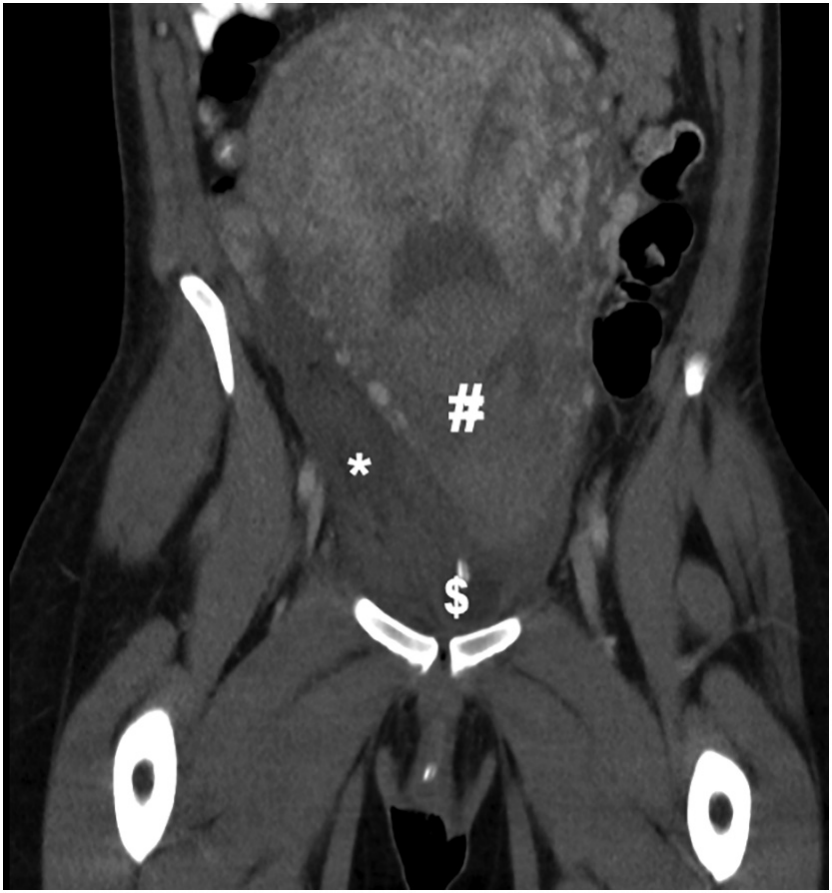
CT scan showing the right broad ligament haematoma*, blood products in the uterine cavity#, and indwelling urinary catheter balloon$.

An urgent computerised tomography (CT) examination of the abdomen and pelvis with intravenous contrast demonstrated two extraperitoneal haematomas without active extravasation. The largest measured 7 cm × 8 cm × 12 cm, lying paravaginally and extending superiorly into the supralevator and extraperitoneal space, displacing the bladder, rectum and uterus [Fig. 1]. The second haematoma was located in the extraperitoneal space, underlying the right broad ligament and measuring 12 cm × 2.8 cm × 6 cm on axial CT [Fig. 2].

An initial plan was made to proceed to theatre for examination under anaesthetic and consideration of incision and drainage of the haematoma but the patient's blood pressure improved to 100/50 mmHg and heart rate to 60 bpm. Given the location of the haematoma and the patient's haemodynamic stability, a decision was made for conservative management with ongoing fluid resuscitation, transfusion of one unit of packed red blood cells, analgesia and commencement of intravenous antibiotics to prevent secondary infection of the haematomas.

**Fig. 3:**
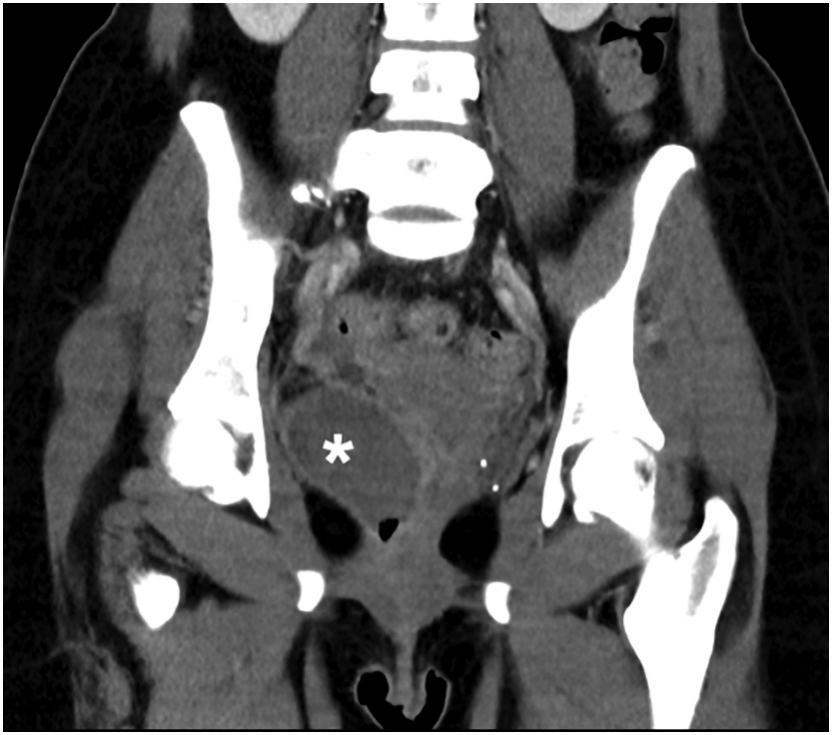
CT scan 2 weeks postpartum, demonstrating reduced paravaginal haematoma*.

The patient's pelvic and rectal pain improved significantly day one postpartum and resolved by discharge on day three postpartum. She completed a two-week course of oral cephalexin and metronidazole and remained well postpartum. Repeat CT of the abdomen and pelvis demonstrated partial resolution of the two haematomas, the largest measuring 6.3 cm × 4.6 cm × 4.5 cm on repeat imaging (Fig. 3). The patient was reviewed on day 20 postpartum and was well, with no perineal bruising or palpable vaginal wall haematoma and a haemoglobin level of 10.2 g/dL, up from 9.4 g/dL at the time of discharge.

## 3 Discussion

While small paravaginal haematomas are common postpartum, only a few cases of paravaginal haematomas causing severe postpartum haemorrhage have been published and a conservative approach in managing these when diagnosed immediately postpartum has not previously been described.

Commonly, the approach to paravaginal haematomas in the extraperitoneal space has been incision and drainage if the haematoma is accessible vaginally. The failure of incision and drainage in three cases, with the need for subsequent transcatheter arterial embolisation (TAE) described by Baruch et al. [3] and Villella et al. [4] and laparotomy in one by Singh et al. [5], highlights the potential difficulties in accessing and achieving haemostasis in this large potential space. Laparotomy and radiologically guided drainage have also been described as primary approaches [6,7].

Only two previous case reports describe conservative management but both were delayed presentations, diagnosed on day 3 postpartum by CT and were not appreciable clinically [8,9].

The use of TAE by interventional radiologists is effective as a uterus-sparing method for severe postpartum haemorrhage, has been shown to be safe in large case series and has clear advantages over surgical intervention in selected patients, such as those with coagulopathy [10,11]. With few complications, including bleeding at arterial puncture sites and postembolisation syndrome, the main limitation on its use is its availability, whether that be in time-sensitive emergencies, out-of-hours or at smaller centres not serviced by interventional radiology [10].

While there may be pressure to intervene in cases where the paravaginal haematoma causes significant pain or severe postpartum haemorrhage, doing so may present its own challenges and risks. Surgical intervention carries the risk of infection, a general or spinal anaesthetic and further haemorrhage, particularly if the haematoma has tamponaded itself, whereby decompression may perpetuate the haemorrhage. It may also present technical challenges in accessing and closing the remaining potential space. This is demonstrated in a case described by Singh et al. [5] of failed evacuation transvaginally followed by laparotomy to further evacuate haematoma from the retroperitoneal space and control bleeding. The capacity for paravaginal haematomas to extend superiorly into the extraperitoneal space is a key contributor to this complexity. Given this, preoperative CT with intravenous contrast could be used in selected cases to help guide management and prevent operators from misadventure during evacuation of what appears to be an easily accessible haematoma on clinical examination. Fig. 1, Fig. 2 show how far a haematoma may extend despite being palpable vaginally.

Our case validates the use of a conservative approach for even large paravaginal haematomas in patients who can be stabilised with IV fluids and blood products. These paravaginal haematomas are likely to self-tamponade and analgesia is then the key focus of management.
